# Exploring adolescents and young people’s candidacy for utilising health services in a rural district, South Africa

**DOI:** 10.1186/s12913-019-3960-1

**Published:** 2019-03-28

**Authors:** Busisiwe Nkosi, Janet Seeley, Nothando Ngwenya, S. Lerato Mchunu, Dumile Gumede, Jane Ferguson, Aoife M. Doyle

**Affiliations:** 1 Africa Health Research Institute, Nelson R. Mandela School of Medicine, 3rd Floor, K-RITH Tower Building, 719 Umbilo Road, Congella, Durban, KwaZulu-Natal 4001 South Africa; 2 London School of Hygiene & Tropical Medicine, Keppel Street, London, WC1E 7HT UK

**Keywords:** Candidacy, Health service utilisation, South Africa, Vulnerability, Young people

## Abstract

**Background:**

We use the ‘candidacy framework’ to describe adolescents’ and young people’s (AYP) experiences of health services in a rural KwaZulu-Natal district, South Africa.

**Methods:**

A qualitative approach was used including group discussions, in-depth and key informant interviews with a purposive sample of AYP (*n* = 70), community leaders (*n* = 15), school health teams (*n* = 10), and health service providers (*n* = 6).

**Results:**

Findings indicate tacit understanding among AYP that they are candidates for general health services. However, HIV stigma, apprehensions and misconceptions about sexual and reproductive health, and socio-cultural views which disapprove of AYP pre-marital sex undermine their candidacy for sexual and reproductive services.

**Conclusion:**

Consideration and understanding of the vulnerabilities and reasons AYP exclude themselves will inform interventions to address their health needs. AYP’s participation in the design of health services will increase their acceptability and encourage uptake of services.

**Electronic supplementary material:**

The online version of this article (10.1186/s12913-019-3960-1) contains supplementary material, which is available to authorized users.

## Background

Young people’s life context and their relationships with healthcare providers shape their health seeking behaviours and the extent to which they prioritise their health. In many African countries, challenges in persuading adolescents and young people aged 10–24 years (AYP) to engage with health services have been reported [1–3]. Young people’s social position based on age, gender, marital and socio-economic status can exacerbate marginalisation and vulnerability to poor health. Efforts to improve health outcomes by scaling up youth-friendly services have not yet achieved the intended results [3–7]. The utilisation of preventative and treatment services among AYP remains low, presenting a significant public health problem [4, 5, 8, 9].

In South African studies on health service utilisation among AYP, the potential for HIV stigmatization at the point of delivery, and other health system factors, continue to pose challenges and limit AYP’s uptake of health services. These factors, which include distance to health facilities and long waiting times, are compounded by staff shortages and negative healthcare provider attitudes [3, 10–12]. Improving our understanding of the vulnerabilities and likely points of exclusion for AYP who are in contact with the health system will inform improvements in healthcare provision and should lead to improved health outcomes.

We used the Candidacy Framework to describe the experiences of AYP’s candidacy for healthcare services in a rural district in KwaZulu-Natal, South Africa. The Candidacy Framework, described below, was useful in guiding our analysis by allowing us to go beyond conventional health service utilisation theories, and explore how AYP’s position in society and healthcare systems influence their decision-making in health service utilisation.

### Theoretical underpinnings

The Candidacy Framework was constructed to investigate the process of accessing health care by vulnerable people in the United Kingdom [13]. The characteristics of the seven stages of the Candidacy Framework are shown in Table 1. The framework has been modified and used on a range of topics including intersectionality and utilisation of other public services beyond the health sector [14–21]. Studies in many sub-Saharan countries, and globally, show that young people are vulnerable to a range of health problems, social and economic hardships. The Candidacy Framework is helpful in improving our understanding of how AYP may receive compromised services through an interacting set of individual, cultural and organizational factors.

**Table 1:** Characteristics of the seven stages of the Candidacy Framework

Stages of candidacy	Description of stages
Identification of candidacy	The process by which individuals come to view themselves as legitimate candidates for particular services
Navigation of services	Knowing how to interact with appropriate services in relation to identified candidacy
Permeability of services	Includes the level of explicit, implicit gate-keeping within a service and the complexity of its referral systems referring to the ‘cultural alignment’ between users and services
Appearing at services and asserting candidacy	The actions that individuals must take to assert their candidacy in an interaction with a healthcare professional
Adjudication by professionals	Candidacy, as expressed by service-users, is validated or otherwise by healthcare professionals which influences subsequent service offers
Offers of, resistance to, services	Emphasises that follow-up services may be appropriately or inappropriately offered and that these may or may not be acted upon by service-users
Operating conditions and local production of candidacy	This incorporates factors that influence decisions about subsequent service provision (e.g. the resources available for addressing candidacy) and the kinds of contingent relationships that develop between professionals and service-users over a few encounters

Source: [16]

Unlike health service utilisation theories which tend to view formal entry into healthcare services as the initial point for consideration, the Candidacy Framework provides a lens on how access is negotiated, determined, and enabled for vulnerable and marginalised people [14, 19, 20, 22]. The utilisation of healthcare services is not based on a single decision but multiple actions occurring over time and access is continually negotiated, subsequently, an individuals’ beliefs about their health condition affects whether they use formal healthcare services. The candidacy lens helps to capture these complex vulnerabilities, as well as the processes that influence how individuals and healthcare systems come to define eligibility for healthcare [13, 19, 21]. The primary aim of the study was to explore health service demand and utilisation among AYP, aged 10–24 years old, as well as to identify potential areas for intervention, in a rural district in South Africa. To the best of our knowledge, no studies have used the framework to explore AYPs access to health care.

## Methods

### The study setting

**Fig. 1:**
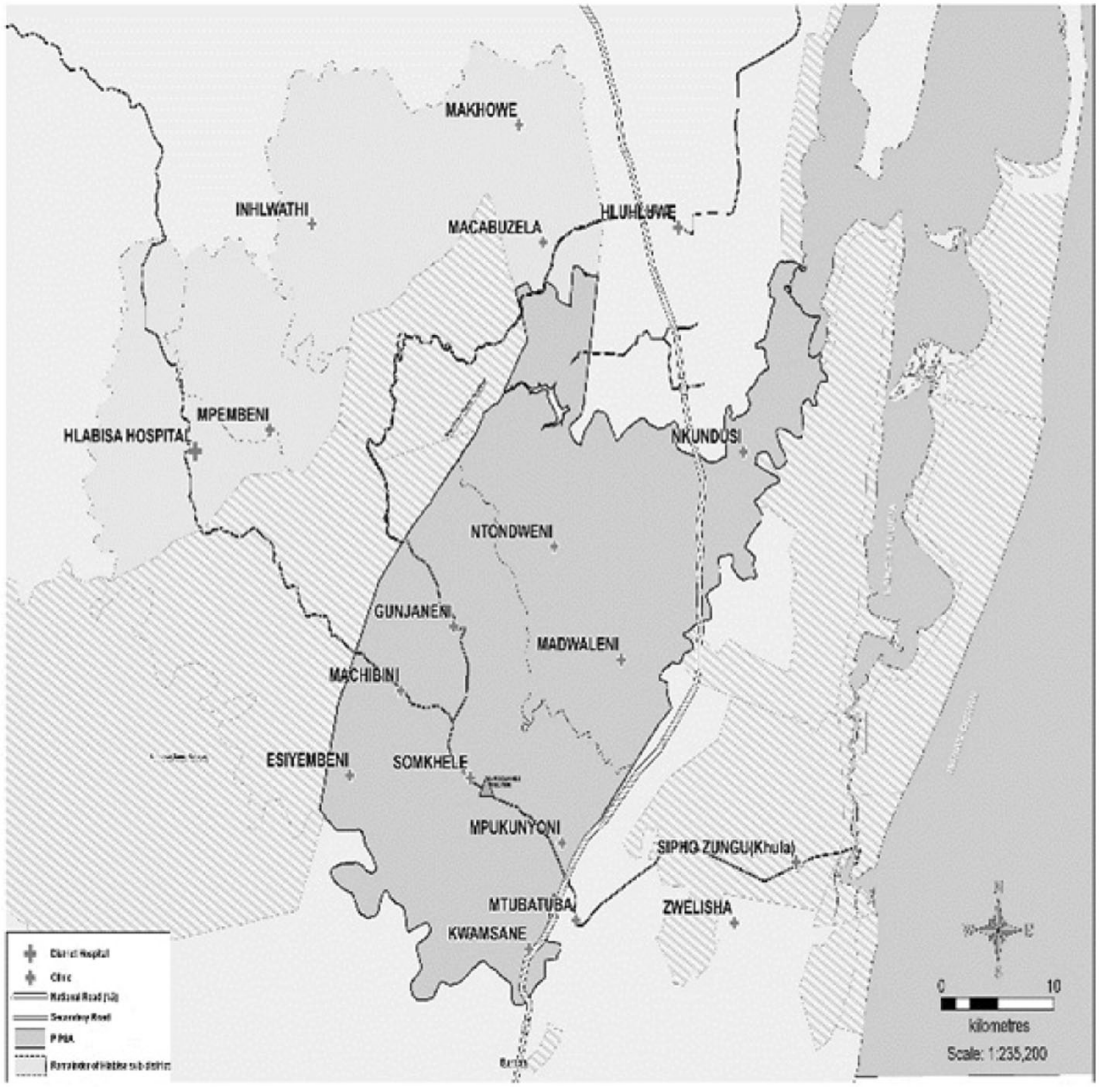
Population Intervention Programme Surveillance Area, (PIPSA), (used with permission) Africa Health Research Institute. Map of the Population Intervention Population Surveillance Area (PIPSA), boundaries in which the Africa Health Research Institute operates (in grey); the rest of Hlabisa sub-district (in light grey). The distribution of the clinics in which the study was conducted are shown in crosses, and Africa Health Research Institute in a triangle

The Health Services for Young People (HSYP) study was conducted in the Population Intervention Programme Demographic Surveillance Area (PIPSA) of the Africa Health Research Institute (AHRI), in uMkhanyakude district, South Africa (see Fig. 1). There are approximately 27,000 AYP (10–24 years old) resident in PIPSA [23]. In this population, a high prevalence of HIV and sexually transmitted infections (STIs) has been reported among young people aged 15–24 years (chlamydia 8.1%, and herpes simplex virus-2 22,2%). HIV prevalence is higher among young women (19.0%) compared to young men (5.6%) [24]. Data for the HSYP study was collected between January and June 2017 using quantitative and qualitative methods. In this paper, we report findings from the qualitative study using COREQ, (Consolidated criteria for reporting qualitative research) a 32 item checklist for reporting qualitative research [25].

### The health care system

South Africa’s health system comprises the public and the private sectors, with access to private healthcare services depending on an individual’s ability to pay. While maternal and reproductive health services are free for pregnant women, children, and senior citizens, primary healthcare provision for the general population is means tested and some clients pay user-fees. The majority of patients access healthcare services through the public sector District Health System (DHS), a decentralised, government mechanism for healthcare provision within a primary health care approach [26]. In uMkhanyakude district, there are five district hospitals, 251 primary care clinics, and 17 mobile clinics servicing a population of 625,846. Doctors from all 5 hospitals visit primary care clinics at least once a month as there are no doctors allocated to the clinics. The clinic-based services are limited in their ability to conduct community outreach and, being focused on curative aspects, are often inadequate regarding prevention, health promotion and rehabilitation services [26]. Ward-based outreach teams (WBOTs), critical to South Africa’s Primary Health approach, do not exist in uMkhanyakude district. WBOTs are designed to bring prevention and health promotion services to the communities based on geographic catchment areas. Limited resources- both human resources for health and medical equipment, structural and health systems factors constrain the implementation of National Youth Friendly policies aimed to reach and address AYP’s health needs [5, 27, 28].

### Community entry

The HYSP study was initially presented to the Community Engagement Unit (CEU) at AHRI. The CEU serves as the community entry point and liaises between communities and AHRI to ensure that research is culturally sensitive and aligns with human rights and ethical standards. Subsequently, the study aims, and objectives were presented to the Community Advisory Board and community leaders to ensure cultural appropriateness.

### Data collection

We used a qualitative approach to conduct group discussions (GDs), in-depth interviews (IDIs), and key informant interviews (KIIs) with AYP, community leaders, school health teams, and health service providers. Participants were recruited face to face in the schools, community and health facilities using purposive sampling. We explained the study aims and rationale during recruitment, and during the consent process.

NN and SLM, researchers experienced in health and social science research (PhD and MA), provided oversight of data collection process. A team of ten fieldworkers, five males and five females, trained in social science and qualitative research methods, conducted the interviews. LSM conducted weekly debrief sessions with the fieldworkers and reviewed interview summaries and field notes in order to capture and reflect on the interview context. NN and BN attended some but not all the debrief sessions.

Interview guides were piloted with 19 participants: health facility staff (*n* = 5), community stakeholders (*n* = 6), school health team (*n* = 2), AYP exit interviews (*n* = 3), accompanying parent (*n* = 1), and AYP (*n* = 2). Data obtained during the pilot were excluded from the analysis. We successfully recruited 101 participants. Table 2 shows data collection methods and the study sample. There were 10 refusals, citing lack of time (*n* = 8), and concerns that the study would encourage undesirable sexual behaviours among AYP (*n* = 2) as reasons for refusal.

**Table 2:**
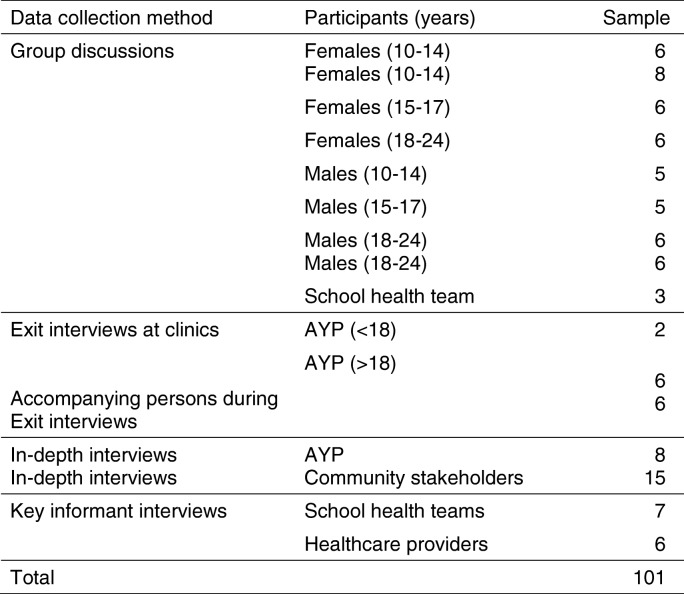
Data collection methods and study sample

Face to face interviews using semi-structured interview guides (see Additional file 1: Study topic guide) were conducted in parallel with community spiral walks to map community resources, to understand the broader community context, and to identify the health needs of AYP. Exit interviews were conducted with AYP and caregivers (where applicable) respectively, after presenting at a health facility. Eight AYP participated in IDIs. Eight GDs were conducted among age (10-14y, 15-17y, 18-24y) and gender stratified groups of AYP (see Table 2), learners and out of school youth, and AYP across peri-urban and rural settings enabling us to capture a wide range of views with differing experiences regarding health needs, health services availability, health service utilisation and barriers to health service utilisation. Similarly, IDIs with community leaders (*n* = 15) and KIIs with health providers (*n* = 8), and school health teams (*n* = 6) added depth on complex socio-cultural phenomenon, barriers and health services utilisation by AYP. Pre-defined interview questions exploring the study aims and objectives covered the following topics: community issues that affect AYP, health services available for AYP, health utilisation among AYP, AYP’s health needs and barriers to health utilisation.

Interviews were conducted at the participants’ homes, and at schools and health facilities. No one else was present during the interviews. Interviews, conducted in a local language isiZulu and/or in English, were audio recorded and lasted between 45 to 120 min. IDIs, GDs and KII occurred in one intensive phase of fieldwork, and it was therefore not possible to accurately assess the data for saturation prior to analysis of the translated transcripts. We did not conduct repeat interviews.

We did not return the transcripts to the participants for comments. However, following initial analysis, stakeholder workshops were conducted separately with health facility staff and AYP to enable peer validation of findings and collective reflective learning.

### Ethics approval and consent to participate

The Biomedical Research Ethics Committee, University of KwaZulu-Natal approved the study (approval number, BE472/15) and the Ethics of the London School of Hygiene and Tropical Medicine, (Ref 11,092). Written informed consent was obtained prior to data collection. For participants < 18 years in addition to obtaining their assent, their parents or a caregiver (where applicable) gave written consent.

### Data analysis

Transcribed data were translated into English and iteratively analysed for themes using content analysis. A deductive and inductive organising framework was used to organise the data and to develop codes and themes. Examples of the coding process are shown in Table 3, and the synthesis of data coding and categories in Table 4. After familiarisation with the interviews, three researchers (BN, SLM, NN) and fieldworkers conducted initial open coding independently. These were reviewed by two co-authors (JS and AD). Themes were determined by the content of the interview guides combined with an inductive development of codes as they emerged from the data [29]. BN, NN and SLM developed a working analytic framework, which was reviewed by JS and AD.

**Table 3:** Example of coding process

Meaning unit	Code	Subcategory	Category
I would say young people take longer to go to the clinic…they may be sick with the virus [HIV] and not know until they get too sick and are forced to [go to] the health facility… Community member, 58 years	Delay to seek health care	Health utilisation	Young people’s lives
They only go to the clinic if they are forced to, like for example, if they are pregnant. They then have to go to the clinic and when they do it is like they are compelled to do a blood test. Community member 43 years	Delay to seek health care	Challenges	Young people’s lives
It [poverty] affects females a lot because you find that if one does not have an ID [identification document], she would end up living with a guy because he provides everything for her. Female exit interview, 22 years	Unemployment and poverty	Challenges	Community characteristics
AYP do not want to access their HIV treatment at the clinic because they will be seen… It is because people who are HIV-positive have pride and they fear that they will become a subject of gossip ‘that you see that proud person, he is infected’… They [patients at the local health facility]] are also identified by files, [colour coding system used for patient files]. The file for a minor sickness, and HIV-positive person are [coded] differently… GD females 18–24 years	Stigma, HIV	Challenges	Health characteristics

**Table 4:** Synthesis of data: coding and categories

Categories	Community characteristics		Young people’s lives			Health characteristics		
Sub-categories	Challenges	Social support	Challenges	Health needs	Health utilisation	Health system factors	Youth friendly services	Modes of health delivery
Codes	Unemployment Poverty Crime and substance abuse Lack of recreational facilities	Family, friends, teachers	Unplanned pregnancies Delay to seek health care Unprotected sex HIV Risky behaviours Vulnerability Embarrassment Fear	Real and perceived needs Specialised health services Health services in the community	Delay seeking health services Traditional health practitioners Primary health services HIV testing and treatment	Distances to the clinics Transport costs Overcrowding Accessibility and availability of health services Health staff attitude Health clinic opening hours Limited health services	Happy hour Youth champions	Referrals Hospitals Schools NGOs

Once the themes were identified, connections across themes were sought through ordering and re-ordering using NVivo 10. The first author (BN) developed a codebook to reflect the themes and topics covered during the interviews. Using an iterative process, the study team revised and modified the codebook to reflect emerging themes throughout the analysis process. Differences and discrepancies were discussed until a consensus was reached. A spreadsheet was used to generate a matrix and the data including references to participants quotations were charted into the matrix. We then explored these themes with reference to the framing of candidacy. A descriptive narrative approach is used to present the findings, along with participants quotes to support and illustrate these findings.

#### Trustworthiness

Triangulation of findings from different data sources (AYP, community leaders, health providers and school health teams), and data methods (GDs, IDIs, KIIs, community mapping) were used to increase validity of our findings. Field notes, debrief sessions, and peer reflections between the field team and authors were conducted to discuss codes and categories generated from the data. Translation of interviews from isiZulu to English was performed by the fieldworkers, checked by SLM, NN and BN, and were ultimately reviewed by the authors. An audit trail helped to validate the coding process and data analysis.

##### Findings

In this section, we first focus on AYP contextual situation followed by their experiences in accessing health care using the candidacy lens. The main themes identified were AYP’s lives, community characteristics, and health characteristics. We then mapped our key themes into the stages of the Candidacy Framework.

### Adolescents and young people’s contextual experiences

AYP included learners from primary and secondary schools, school dropouts, and unemployed youth. Most of them lived with their parents or relatives. Participants described the community as beset with crime, poverty, unemployment, alcohol and substance abuse. These social ills coalesce to reinforce vulnerability and risky behaviours that often lead to HIV infection and/or unplanned early parenthood and interruption of their education. While younger women often drop out of school, and must cope with unplanned pregnancies, young men dropped out of school primarily due to alcohol and substance abuse.

Interviews revealed that although HIV is universally feared, behaviours that increased the risk of HIV such as unprotected sex, and having multiple partners were common. Complex sexual relationships between young women and older men often include monetary and material benefits or exploitation by older men, and increased risk to HIV. Poverty and lack of prospects for earning a living left young women vulnerable and led to exploitation by older men. A female exit interviewee (22 years) stated:

It [poverty] affects females a lot because you find that if one does not have an ID [identification document], she would end up living with a guy because he provides everything for her. However, if she had an ID, she would be able to support herself. So, she may end up staying with a guy because there is no way of making a living… and she might be at risk for abuse…

### Identification of candidacy

The interviews revealed that AYP were more likely to identify themselves as candidates for primary care services through tacit understanding, and/or through experiencing a series of health problems. The interviews indicated that many AYP delayed seeking health care until they were very ill, and the severity of symptoms became a key motivator for seeking health care. A female community member (58 years) stated:

I would say young people take longer to go to the clinic…they may be sick with the virus [HIV] and not know until they get too sick and are forced to [go to] the health facility…

Candidacy was also expressed through prior appointments for consultation with specialist care providers, and follow-up visits if there was no improvement after the initial visit.

Exit interviews with female and male participants showed that AYP’s candidacy for HIV testing tended to be tied to antenatal care visits among females and STIs for males respectively. This view was expressed during an IDI by a community member (43 years):

They only go to the clinic if they are forced to, like for example, if they are pregnant. They then have to go to the clinic and when they do it is like they are compelled to do a blood test.

We found that it was usual practice for AYP to claim candidacy with multiple providers, such as traditional healers, pharmacists and private general practitioners*.* Younger males particularly reported consulting traditional healers for STI treatment, and later visited health facilities when their symptoms persisted. The participants discussed being embarrassed to be seen at clinics. Some of the participants preferred consulting with health facilities outside their catchment areas, where they are not known, because of privacy concerns.

The lack of privacy, and stigma associated with HIV was a barrier to many AYP utilising HIV services, and GD participants (females, 18–24 years) indicated that AYP:

…do not want to access their HIV treatment at the clinic because they will be seen… It is because people who are HIV-positive fear that they will become a subject of gossip ‘that you see that proud person, he is infected’… They [patients at the local health facility] are also identified by files [colour coding system used for patient files]. The file for a minor sickness, and HIV-positive person are [coded] differently…Files for HIV-positive people are pink and for minor sickness are blue…That is the reason why most of the people access their treatment at [name of facility out of the local catchment area].

Fear of stigma and potential scolding by health providers’ undermined AYP’s candidacy for HIV services. This view was often repeated by many AYP, and male GD participants (10–14 years) stated:

People are afraid to collect their treatment, and they miss their dates because they fear that nurses [will] shout at them based on their health issues [HIV status] in public.

Community stakeholders reiterated in their interviews the ways in which stigma acts as a barrier in health facilities. Community interviews suggested alternative ways that AYP can access HIV treatment whilst ensuring their privacy, as stated during an IDI, with a female community member (41 years):

In clinics, there are numbers classifying and people who enter specific consultation rooms, because as people we are judgmental. If you enter the clinic, they will say you have this kind of disease. Other people, they don’t want to disclose. I would say if it can happen that there are places where people can collect their treatment privately, because there are people who are scared to visit clinics.

We also found that AYP’s knowledge about HIV, other STIs and sexual and reproductive behaviours was patchy. There were misconceptions about the use of contraceptives and fertility which influence AYP’s uptake of the contraceptives. A female participant (22 years) stated the following during an exit interview:

They say you are not allowed to use contraceptives because you won’t be able to conceive when you are ready to have a child, since the injection birth control method takes longer to leave a woman’s body… it takes longer time to return to fertility if you have been using the injection birth control method. For example, if I want to have a child this year (2017) it won’t be possible but maybe in 2018 I can be able to conceive, because it takes time for it to work its way out.

### Navigation of health services

In navigating the health systems, systemic factors such as distances to health facilities, transportation costs, and long waiting queues were seen as influencing AYP’s readiness to seek health care. Narratives with GD females (10–14 years) showed that:

…the clinic is too far for them, about 5–6 km [about 3.7 miles] away and you have to get up at 5 am to get to the clinic by 7 am. Those who have money travel by taxis which costs them R15 [about $1.15] and a return trip is R30 [about $2.30]. If you do not have money you walk. Even so, you only get assistance in the late afternoon around 3 pm, and that means you must spend the whole day at the clinic.

Although these system factors occasionally prompted AYP to seek alternative providers such as traditional healers, pharmacists and private general practitioners, the AYP ultimately returned to public health clinics.

### The permeability of services and appearing at services

Specialised surgical, clinical, counselling, therapeutic and other services were not readily available or were limited to certain days, and participants were often referred to the district hospitals. Interviews revealed that many AYP experienced intense emotional distress and lived in an area with a high prevalence of alcohol and substance abuse, yet discussions about the provision of mental health service were limited. This was noted during GD with females (18–24 years):

… we don’t have those kind of services [mental health services] here you must travel far if you want that service. Like the place where a person would go to if he abuses alcohol… in rehabs, there is no place like that here.

Health services were generally perceived as inadequate and often unresponsive to their health needs, a concern noted during a GD with females (18–24 years):

It is just that this clinic facility does not have medication and is overcrowded. You return home by sunset when they close because sometimes they close when you haven’t received any medical care service.

For some of the participants, this was frustrating, and it meant making numerous trips to the health facilities either to collect medicine or to consult with specialist healthcare providers.

### Adjudication by professionals, offers of and, resistance to services

AYP’s claim to services is ultimately validated by health providers’ perspectives which influenced subsequent service provision. Narratives during the interviews and the stakeholder workshops underscored a misalignment between healthcare providers’ religious beliefs and the provision of abortion services, and often revealed disapproval of contraceptive use among young unmarried women. Although adolescent girls from age 12 have the right to seek an abortion and family planning services in South Africa, the constant use of the language “killing of an innocent soul” by some healthcare providers discouraged AYP from seeking abortion services [27, 28].

Similarly, local moral dimensions which sanction premarital sex influenced healthcare provider roles in adjudicating SRH services. AYP felt that they were judged morally during antenatal care visits. Narratives during a GD with females (10–14 years) revealed:

When pregnant young females visit the clinic, the nurses would ask them why they engage themselves in things that are inappropriate for their age…

These views made AYP feel embarrassed and reluctant to express their problems as a female AYP (23 years) said: “young people are scared, we do not like to say I have a problem”.

### Operating conditions and local production of candidacy

Strategic level factors that influence subsequent health service provision either enabled or denied AYP’s access to health care. We found that local realities, such as inadequate health staff and poorly resourced facilities, undermined provision of health services. Narratives during GD with females 18–24 noted:

So, when [adjacent] communities visit this clinic, it runs out of medication. There are no emergency services … and mobile clinics do not have medication.

Participants raised concerns about the organisation of health services delivery and pointed out that the lack of adequate space in health facilities and mobile clinics compromised their privacy and confidentiality. They noted that they were sometimes made to wait outside the building while waiting for a consultation because of overcrowding. These factors discouraged healthcare service utilisation among AYP. Lack of clarity in policy compounded implementation of youth-friendly services. A healthcare KII reported:

Policy should indicate who is responsible for which parts of the policy for example, who is responsible for involvement of youth in the running of a youth-friendly policy in the clinic.

Alternative modes of service delivery available for AYP at school through NGOs were limited to providing health education and facilitating referrals between the schools and health facilities. The referral system was implemented inconsistently, depending on individual clinics and healthcare providers’ workloads.

In areas where youth friendly services are implemented, reports by AYP and healthcare providers were positive, however study participants acknowledged that their provision can be patchy and inconsistent. Healthcare providers trained to provide user-friendly services to support AYP, locally known as Youth Ambassadors, were constrained in fulfilling their roles, because of the many pressing responsibilities and priorities, a situation compounded by staff shortages and overcrowding. In these conditions, health staff often focused on ‘pushing the lines*’* to consult as many patients as possible. The ‘happy hour’, an extension to operating hours designed to mainly afford AYP the opportunity to access health care, was not implemented and did not address the distances that AYP have to travel to health facilities. The safety of AYP and healthcare providers’ lives were also at risk especially in the evening because of widespread crime in the area. Despite the weaknesses and limitations, narratives with AYP suggested that they were unequivocally enthusiastic about the Youth Ambassador model, and they felt that it provided them with an outlet to talk about sexual behaviour and non-health issues.

## Discussion

The candidacy lens helped us to capture the complex vulnerabilities and processes that come to define eligibility for healthcare as well as the utilisation and uptake of health services among AYP in a rural district in South Africa.

### AYP’s contextual experiences

Our findings show that AYP face multiple aspects of vulnerabilities including age relative to the healthcare providers, gender, poverty, stigma, and rurality, which undermine AYP’s health utilisation [30]. We also found that the socio-cultural perspectives and moral dimensions which are more pronounced in HIV and SRH services with the younger unmarried AYP, make them feel embarrassed and discouraged them from utilising these services [10, 13, 19, 31]. It is therefore important to consider the hierarchal nature of the vulnerabilities and how this has potential to marginalise AYP and subsequent health seeking behaviours.

### Identification

We show that most of AYP tacitly see themselves as candidates for primary health services but were apprehensive about HIV and SRH services. This fits with the view that identification of candidacy for a particular set of services is socially constructed and dynamically influenced by one’s past encounters with the health services [13, 16]. We also show that severity of symptoms for illnesses including HIV or pregnancy prompted AYP to seek health care, consistent with literature on vulnerable groups and access to health care [14, 17, 28]. We also found misconceptions about SRH among the AYP, a finding which has been reported in other African countries [31, 32], and among female students in London, United Kingdom [28]. Therefore, accurate knowledge, improving AYP’s ability to interpret and evaluate symptoms, and the need for routine healthcare utilisation are essential to claim candidacy and appropriate care [28].

### Navigation

As widely reported in other studies [14, 30] we show that travel costs to the clinics, access to a range of services and information, and long waiting queues posed challenges for many AYP. Few participants received financial support from families to enable access to care, including private General Practitioners. Some of the AYP, mostly males, sought alternative care from traditional health practitioners instead of dealing with gate-keeping in the healthcare system, consistent with other South African studies [33, 34].

### Permeability of services and appearing at services

Specialist services, such as abortion and dental care, were either limited or not available, and participants were referred to the district hospital. Limited range of services exacerbate vulnerabilities and add transportation costs, and time spent travelling to seek services. Integration of primary care and HIV services is both feasible and it encourages utilisation of healthcare services in areas with limited resources and high burden of diseases such as Haiti [35].

### Adjudication by professionals, offers of and, resistance to services

Consistent with the literature, we show that health providers play a central role in sifting potential users at the point of access to ensure ‘appropriate’ uptake of particular services in other sub-Saharan African countries [29, 36–38]. Pathologizing AYP’s sexual behaviours and use of contraception discouraged many AYP from seeking SRH services from the clinics. Other studies found that many nurses in South Africa think it is wrong for AYP to have sex, and that this affected their clinical judgement [10, 31]. This highlights the importance of training and supporting healthcare providers to be sensitive to these issues and lessen inappropriate adjudication based on illness category [10, 33].

### Operating conditions and local production of the candidacy

Our findings on the operating conditions and local production of the candidacy stage are similar to the ‘street-level’ bureaucracy literature which incorporates factors which might void or validate candidacy- policy imperatives, contingent relationships between AYP and health providers, available resources for addressing candidacy as well as health providers’ discretionary decision making [16, 31, 32]. Despite efforts to make health services youth friendly, the public health system is largely governed in a hierarchical, authoritarian style, which is counterproductive. Like other studies, our findings show that health care providers tended to impose their values upon AYP regarding provision of contraceptives to younger women, and termination of pregnancy, blurring clinical obligations [28, 30]. Structural factors and health providers’ discretionary decision making were demonstrated by their inability to consistently implement elements of youth friendly services such as the happy-hour, and Youth Ambassadors. Faced with staff shortages, and overcrowding, health-providers pragmatically tended to adopt a coping mechanism and focused on attending as many clients as possible. Our findings suggest that integrating HIV and SRH into primary health care services has the potential to encourage the uptake of HIV and SRH services in settings where stigma and socio-cultural norms undermine AYP’s candidacy to seek health services. Strengthening alternative modalities of health delivery through increased collaboration with NGOs, schools, community caregivers, and WBOTs may help to reduce overcrowding in the clinics and provide AYP with more healthcare options. Studies show that community-based services provided by community caregivers and NGOs, promote health care utilisation by increasing access in remote areas, and by playing a mediating role between the formal health system and vulnerable populations [33, 39–42].

### Strengths and limitations

Our study explores health service needs and utilisation for a wide range of health concerns affecting AYP and is not limited to HIV and SRH. We provide insights on how AYP’s lives, socio-cultural environments and health systems work together to reinforce or constrain AYP’s claim to health services. In the context of well-established National youth-friendly health service policies, our findings point to unremitting structural factors which constrain effective health service provision, especially in the rural areas. One of the study limitations was the limited participation of younger AYP exiting the health facilities because of the difficulty in obtaining parental consent for those younger than 18 years to participate in the study. The requirement for parental consent for research contrasts with the right to seek health care without parental consent from age 12 [33, 34]. We did not directly explore how AYP understand or define youth friendly services, however, there was a level of understanding of the elements of youth friendly services particularly among the learners. Our findings are based, therefore, on comments made in the context of discussing health service provision more generally.

## Conclusion

The findings show that while AYP tacitly see themselves as candidates for general health services, they were apprehensive about requesting HIV and SRH services. Over and above healthcare-providers’ relationships with AYP and health service utilisation, our findings show that AYP are afraid of being embarrassed when seeking health services. Multi-sectoral interventions that target AYP vulnerability should be co-developed with adolescents and other stakeholders to encourage AYP uptake of health services. Working closely with AYP will ensure that modalities such as HIV testing and STI screening are incorporated in ways that are acceptable and which encourages their uptake. Consideration and understanding of the vulnerabilities and reasons AYP exclude themselves will inform interventions to address their health needs and assist in providing more comprehensive and timely healthcare for AYP.

## Additional file

Additional file 1: Study topic guide. Interview guide for the key informant interviews with health providers, school health teams, in-depth interviews with AYP and community leaders, exit interviews with AYP, and group discussions with AYP. (DOCX 15 kb)

## Abbreviations

AHRI: Africa Health Research Institute
AYP: Adolescents and young people
CEU: Community Engagement Unit
GDs: Group discussions
HIV: Human immune-deficiency virus
HSYP: Health Services for Young People
IDIs: In-depth interviews
KIIs: Key informant interviews
NGOs: Non-governmental organisations
PIPSA: Population Intervention Programme Surveillance Area
SRH: Sexual and Reproductive Health
STIs: Sexually transmitted infections
WBOTs: ward based outreach teams
