# Supplementary material for: Exploring adolescents and young people’s candidacy for utilising health services in a rural district, South Africa
Source: BMC Health Serv Res. 2019 Mar 28;19:195. doi: 10.1186/s12913-019-3960-1 (PMC6438017; doi:10.1186/s12913-019-3960-1)
Supplement: Supplementary file 1 — Study topic guide. Interview guide for the key informant interviews with health providers, school health teams, in-depth interviews with AYP and community leaders, exit interviews with AYP, and group discussions with AYP. (DOCX 15 kb) [file 12913_2019_3960_MOESM1_ESM.docx]

**Study topic guide**

**Health service provision for and utilisation by young people (10-24 years) in rural KwaZulu-Natal, South Africa**

Important note: This guide indicates the topics that we plan to cover in our interviews with study participants.

**1. Health facility staff**

h) Services that are offered & level of demand

Is there a particular place where adolescents/young people are provided health services?

What services are available for young people with mental health problems?

What kinds of health problems require referral to a service external to this health facility?

What services do boys/young men access? Is there any demand for circumcision?

Is circumcision offered at this facility? If not, where can young men get circumcised?

What HIV testing services are available for young people?

What support is available for young people who are HIV+ to ensure that they continue their ART?

i) Health services that adolescents would like but which are not available

j) Barriers/facilitators to service provision for adolescents

k) Staff training needs

Have staff received any training on the provision of services to adolescents/young people?

l) Attitudes towards adolescents use of services (esp. SRH)

What services do young people need?

Who should be getting condoms?

What should sexually active young people do if they want to prevent pregnancy?

m) Suggestions on how services could be improved

**2. School health team staff**

a) Services that are offered & level of demand

What services do young men request?

What services do young women request?

What services can you offer a young person who has been a victim of violence?

Do you provide any nutritional advice?

What kinds of health problems require referral to a service external to this health service?

Is there any provision for menstrual hygiene?

What happens if a young man would like to be circumcised?

What support is there for young people who indicate that they are HIV+ to ensure that they continue their ART?

b) Health services that adolescents would like but which are not available

c) Barriers/facilitators to service provision

d) Staff training needs

e) Attitudes towards adolescents use of services (especially sexual and reproductive health)

f) Suggestions on how services could be improved

**3. Health facility exit interviews**

Iinterviews with young people (and accompanying parent/guardian) as they leave the health facility

a) Reason for visit/ presenting condition

What is the main reason that you have come here today?

How long have you had this condition?

Did you delay coming to the health service? Why?

Did you initially seek care from another care provider (private GP, traditional practionner, pharmacy)?

Do you intend to seek care from another provider for this same condition after this visit?

b) Treatment/service received

Do you receive any treatment for the condition that you presented with?

Did you receive any other additional services or information that were not related to the condition that you presented with?

Were you referred to another facility/service?

c) Attitude of health service staff

How did the health worker make you feel?

d) Confidentiality

Who have you told about your visit here today?

Do you think that anyone else will find out? How?

e) Cost of service & travel (if any)

f) Time spent at health service

g) Level of satisfaction with service

h) Suggestions for improvement of services

**4.** **Group interviews with young people in the community**

a) Most important issues concerning young people in their community

b) Most important health issues affecting young people

c) Where do young people go if they seek health services

d) Health services provided

e) Health services needed/desired

f) Challenges to accessing health services

g) Attitudes of health staff towards young people

h) Suggestions for improvement of services (location, timing, services etc.)

i) Communication with parent/carer over health needs

j) Other sources of information on health issues e.g. the internet, media

**5. Interviews with parents/guardians**

a) Most important issues concerning young people in their community

b) Awareness of and attitudes to young people’s health needs

c) Most important health issues affecting young people

d) Views of health services provided in general and for young people

e) Services needed/desired

f) Challenges to accessing services

g) Attitudes of health staff towards young people

h) Suggestions for improvement of services (location, timing, services etc.)

i) Communication with young person over health needs

j) Other sources of information on health issues

**6**. **Interviews with community stakeholders**

a) Most important issues concerning young people in their community

b) Awareness of and attitudes to young people’s health needs

c) Most important health issues affecting young people

d) Views of health services provided in general and for young people

e) Services needed/desired

f) Challenges to accessing services

g) Attitudes of health staff towards young people

h) Suggestions for improvement of services (location, timing, services etc.)

i) Other sources of information on health issues
